# HDL Mimetics Infusion and Regression of Atherosclerosis: Is It Still Considered a Valid Therapeutic Option?

**DOI:** 10.1007/s11886-018-1004-9

**Published:** 2018-06-21

**Authors:** I. Karalis, J. W. Jukema

**Affiliations:** Department of Cardiology C5-P, Leiden University Medical Center, Albinusdreef 2, 2333 ZA, Postbus 9600, 2300 RC Leiden, The Netherlands

**Keywords:** HDL mimetics, Reverse cholesterol transport, Coronary artery disease, Atherosclerosis, CETP inhibitors

## Abstract

**Purpose of Review:**

This review aims to summarize and discuss the recent findings in the field of using HDL mimetics for the treatment of patients with coronary artery disease.

**Recent Findings:**

Following the largely disappointing results with the cholesteryl ester transfer protein inhibitors, focus moved to HDL functionality rather than absolute HDL cholesterol values. A number of HDL/apoA-I mimicking molecules were developed, aiming to enhance reverse cholesterol transport that has been associated with an atheroprotective effect. Three HDL mimetics have made the step from bench-testing to clinical trials in humans and are discussed here: apoA-I Milano, CSL-112, and CER-001. Unfortunately, with the exception of CSL-112 where the results of the clinical trial are not yet known, none of the agents was able to demonstrate a clinical benefit.

**Summary:**

HDL mimetics have failed to date to prove a beneficial effect in clinical practice. Reverse cholesterol transport remains a challenging therapeutic pathway to be explored.

## Introduction

Observational studies in multiple populations indicate a continuous positive relationship among the prevalence of coronary artery disease (CAD) and the blood LDL cholesterol levels that extend well below the ranges seen in Western populations, without any definite threshold below which a lower cholesterol concentration is not associated with a lower risk [1, 2]. Therefore, current practice guidelines concerning high-risk populations are focusing on achieving very low levels of LDL cholesterol, mainly through the systematic use of potent statins. However, despite the efficiency of established therapies, the residual burden of disease remains substantial [3].

Since its discovery by Miller and Miller in 1975 [4], HDL has been associated with a potential protective effect against atherosclerosis. HDL concentrations higher than 75 mg/dl (1.9 mmol/l) were associated with prolonged life (longevity syndromes) and relative freedom from CAD [5]. In the Framingham Study, the risk of CAD was shown to increase sharply as HDL levels fell progressively below the 40 mg/dl (1.04 mmol/l) [6]. The publication of the Helsinki Heart Study in 1987 [7], where a simultaneous 11% increase in HDL and reduction in LDL levels during gemfibrozil therapy were accompanied by a 34% reduction in myocardial infarction rates, raised for the first time the issue of whether efforts to increase HDL levels should be undertaken in patients with CAD and/or dyslipidemia.

### Use of HDL as a Therapeutic Target

Despite the substantial body of evidence from classical epidemiological association studies, HDL cholesterol raising has not been proven to actively reduce cardiovascular event risk or affect the development of atherosclerosis [8]. The argument for lack of causality (for HDL cholesterol) comes from Mendelian randomization analyses on the one hand and the difficulty in demonstrating improved outcomes with therapies that raise HDL cholesterol on the other; therapeutic interventions such as niacin and cholesteryl ester transfer protein (CETP) inhibitors increase HDL cholesterol in patients treated with statins but have repeatedly failed to reduce cardiovascular events with the sole exception of anacetrapib in the REVEAL study, where, however, the beneficial effect was not clearly associated to the rise of HDL alone [9, 10].

**Fig. 1:**
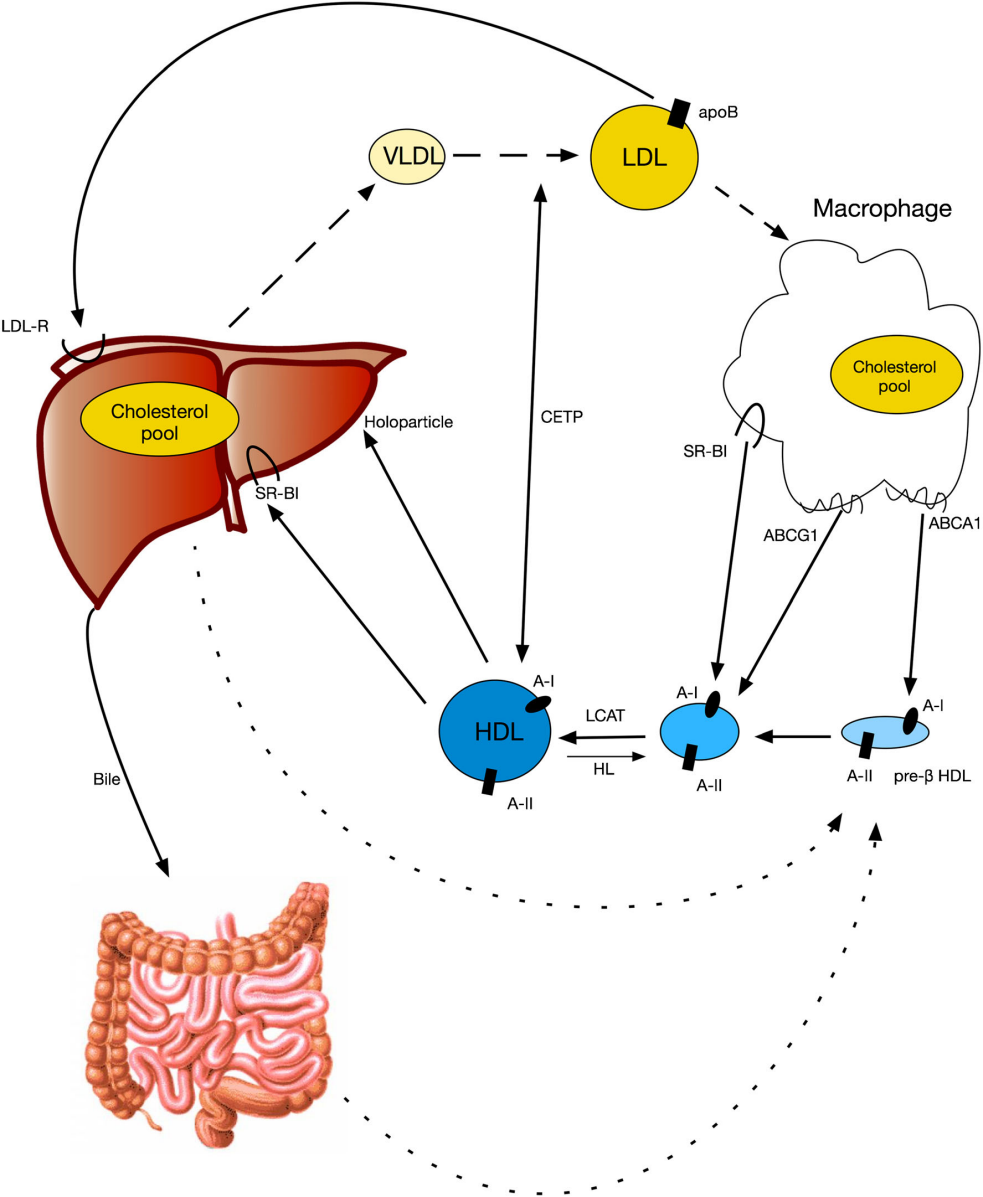
The reverse cholesterol transport pathway. Cholesterol-rich lipoproteins are taken up by macrophages resulting in foam cell formation. From macrophages, efflux of cholesterol follows three pathways: (1) via ABCA1, received by lipid-poor apoA-I/pre-β HDL; (2) via ABCG1, received by more mature spherical HDL particles; and (3) SR-BI mediated or aqueous diffusion. Within HDL, cholesterol is esterified by LCAT, making space on the HDL surface for the uptake of additional free cholesterol. Subsequently, HDL is absorbed by the liver; cholesterol is de-esterified and secreted into the bile. In humans, CETP creates some short of “shunt” among the forward and the reverse cholesterol transport pathways. Therefore, hepatic absorption of apoB containing lipoproteins can also contribute in a way in the reverse cholesterol transport pathway. Finally, the rate of cholesterol absorption from the intestinal lumen has an impact on the amount of foam cell-derived cholesterol that is finally excreted from the body

This discrepancy among the results of the initial clinical trials on the one hand and the pre-clinical data demonstrating atheroprotective properties of HDL on the other shifted focus towards the functional properties of HDL and in particular the stimulation of the reverse cholesterol transport schematically presented in Fig. 1 [11]. We can roughly identify three stages in the reverse cholesterol transport process: (1) cholesterol efflux, where HDL/apolipoprotein A-I (apoA-I; the major protein component of the HDL particles) remove excess cholesterol from cells; (2) lipoprotein remodeling, where HDL undergoes structural modifications with possible impact on its function; and (3) hepatic lipid uptake, where HDL releases cholesterol to the liver, for the final excretion into bile and feces. The development of methods capable of measuring cholesterol efflux capacity gave us a tool where the functional properties of HDL could be measured. It has been demonstrated that sera with similar HDL or apoA-I levels may differ in their ability to promote macrophage efflux, because of differences in the concentration of pre-β HDL [12]. Furthermore, cholesterol efflux capacity proved to be a strong predictor for cardiovascular events, independent of the actual HDL levels [13, 14]. These scientific data reinforced our interest of using HDL as a therapeutic target, aiming however for functional HDL particles/HDL flux rather than simply raising the cholesterol content of the HDL fraction.

Apolipoprotein A-I would represent a promising target. Evidence from both animal and human studies [15–17] suggests an important role of apoA-I in many of the anti-atherogenic properties attributed to HDL. Several therapies imitating apoA-I function have been tested, including administration of full-length apoA-I, mutated variants of apoA-I, and apoA-I mimetic peptides.

### ApoA1 Mimetic Peptides

Apolipoprotein A-I consists of 243 amino acids. Its secondary structure resembles 10 amphipathic α-helices that seem to be important for its efficient interaction with lipids. Over the last 25 years, multiple peptides resembling the amphipathic helices in apoA-I have been engineered and tested as therapeutic agents, mostly in vitro as well as in the lab, in studies with rodents. These agents and the corresponding studies are well summarized in the review article of Stoekenbroek et al. [18^••^]. The in vitro properties of the engineered agents as well as the main findings of the studies performed are summarized in Table 1. To the moment, none of these apoA-I mimetic peptides have been used in a clinical trial in humans.

**Table 1:** ApoA1 mimetic peptides

ApoA1-mimetic peptide	Method of administration	In vitro properties	In vivo results (mice)	Tested in humans
4F	Oral/parenteral	• Anti-inflammatory • Anti-oxidant	• Conflicting results on HDL-mediated protection against LDL induced monocyte chemotaxis [19, 20]	No, development stopped
6F	Oral	• Anti-inflammatory • Anti-oxidant	• Reduction of inflammatory biomarkers • Reduction of aortic lesion area [21]	No studies yet
FX-5A	Parenteral	• Induces cholesterol efflux both through ABCA1 and ABCG1	• Increased cholesterol efflux • Anti-inflammatory properties • Anti-oxidant properties [22, 23]	Not studies yet, possible in the future
ATI-5261	Parenteral	• ABCA1-mediated cholesterol efflux	• Increased reverse cholesterol transport [24]	No studies
ETC-642	Parenteral	• Reduction of pro-inflammatory oxidized LDL • Reduction of sdLDL • Increase of anti-atherogenic pre-β HDL	• Inhibition of atherosclerotic plaque formation [25]	No studies

### ApoA-I-Based Infusion Therapy and Clinical Trials in Humans

Based on animal models where apoA-I overexpression and infusion inhibit plaque formation, it was hypothesized that infusion of apoA-I containing particles, such as lipid-poor pre-β HDL, would also have beneficial effects on plaque stabilization and atherosclerosis regression. As early as in 1990, Badimon et al. [26^••^] demonstrated that treatment of rabbits, who were fed with an atherogenic diet for 60 days in order to induce atherosclerotic lesions, with high-density/very high-density lipoprotein infusions obtained by ultracentrifugation did not only inhibit plaque formation but also reduced the extent of preexisting lesions. Miyazaki et al. [27] could replicate the results of Badimon, treating the rabbits with purified apoA-I. However, it should be noted that the atherosclerotic lesions in rabbits only partly resemble human atherosclerosis. Therefore, testing of these compounds in patients is absolutely necessary in order to determine the (magnitude of) beneficial effects.

In the last few years, three infusible HDL mimetics have made the step from bench testing to clinical trials in humans and will be further discussed here: apoA-I Milano, CSL-112, and CER-001.

i. *apoA-I Milano*. This is a naturally occurring mutation of apolipoprotein A-I, originally documented in a small village in Italy. Carriers of this mutation are characterized by an exceptionally low prevalence of cardiovascular disease despite decreased HDL cholesterol and apoA-I levels [28, 29]. Compared to native apoA-I, apoA-I Milano has been shown to induce more ATP-binding cassette transporter A1 (ABCA1) mediated cholesterol efflux and to exert superior anti-inflammatory and plaque stabilizing properties [30]. These observations have led to the engineering of formulations containing recombinant apoA-I Milano complexed with phospholipids. In a phase II clinical trial, in a small group of patients following an acute coronary syndrome (ACS), administration of such a molecule (ETC-216, Esperion Therapeutics) produced significant regression of coronary atherosclerosis as measured by intravascular ultrasonography (IVUS) [31]. The findings suggested a potential novel strategy for the management of patients with ACS, where the infusion of an agent the first few weeks or months following an acute event would stimulate reverse-cholesterol transport while ongoing therapy with conventional lipid-modulating agents would provide long-term clinical benefits. However, serious side effects prevented further evaluation of this formulation.

Following optimization of the manufacturing process, a newer formulation (not inducing adverse immunostimulation like ETC-216 [32]) called MDCO-216 (The Medicines Company) was tested in a double-blind, randomized, placebo-controlled, multicenter trial (MILANO-PILOT, NCT02678923) with 122 post ACS patients on optimal conventional medical treatment. Following five weekly MDCO-216 infusions, both primary (percent atheroma volume) and secondary efficacy parameters (normalized total atheroma volume and atheroma volume in the most diseased segment) did not differ significantly among the treated group and the control group. The authors conclude that apoA-I Milano failed to demonstrate (on IVUS) incremental regression of coronary atherosclerosis, among patients with a history of ACS and documented angiographic coronary disease, in the setting of contemporary statin therapy (results presented at the American Heart Association Congress, New Orleans 2016). Further development of MDCO-216 has now ceased.

ii. *CSL-112*. Produced by the Commonwealth Serum Laboratories, this is a reconstituted HDL particle, consisting of native apoA-I and phospholipids. A predecessor compound, CSL-111, was tested in the ERASE trial, where IVUS studies on post ACS patients receiving weekly infusions, failed after 4 weeks to lead to significant reductions in atheroma volume or plaque volume compared with placebo but did result in statistically significant improvement in the plaque characterization index and coronary score on quantitative coronary angiography (QCA), thereby suggesting a therapeutic potential, according to the authors [33]. However, administration of high doses of CSL-111 was also associated with hepatotoxicity leading to the development of CSL-112. The later compound, which appeared to be safe in two phase I clinical trials in healthy individuals, was associated with a dose-dependent increase in apoA-I which remained above baseline for approximately 3 days after infusion. A rapid 36-fold increase in pre-β HDL levels was also noted, which was accompanied by increased ABCA1-mediated cholesterol efflux (maximum increase 270% compared to baseline). Multiple infusions of CSL-112 were well tolerated with no evidence of major organ toxicity or immunogenicity [34]. In the AEGIS-I trial that followed, a large 2b clinical trial including 1258 patients with a recent acute myocardial infarction, the aim was to determine the safety, tolerability, pharmacokinetics, and pharmacodynamics of CSL112. The authors of the study concluded that four weekly infusions of CSL112 are feasible, well tolerated, and not associated with any significant alterations in liver or kidney function [35]. Moreover, the study confirmed the ability of CSL-112 to acutely enhance cholesterol efflux. However, the potential benefit of CSL112 in reducing major adverse cardiovascular events in this group of high-risk patients (following an acute coronary syndrome) still remains to be shown in the large phase III AEGIS-II study (NCT03473223) that is currently recruiting patients and is expected to be concluded in 2022.
iii. *CER-001*. CER-001 (Cerenis Therapeutics, France) is a negatively charged lipoprotein complex mimicking discoidal pre-β HDL, consisting of recombinant human apoA-I and a combination of two naturally occurring phospholipids, diphosphatidylglycerol, and sphingomyelin. Preliminary studies showed that, following iv administration, CER-001 can rapidly mobilize large amounts of cholesterol into the HDL fraction. The CHI-SQUARE study [36] was designed to assess the safety and efficacy of CER-001 in the clinical setting. A total of 507 patients with a clinical indication for coronary angiography were included; IVUS and QCA were performed to assess coronary atherosclerosis at baseline and 3 weeks after the last study infusion. Patients were randomized to receive six weekly infusions of placebo, 3, 6, or 12 mg/kg CER-001. Despite the dose-related increase in cholesterol mobilization observed (estimated by the increase in plasma cholesterol after CER-001 infusion), neither IVUS nor QCA were indicative of reduction of coronary atherosclerosis in the treated groups when compared to placebo. However, a post hoc analysis demonstrated that in patients with a baseline percent atheroma volume more than 30%, infusions of CER-001 at a dose of 3 mg/kg induced the greatest atheroma regression, while higher concentrations of CER-001 did not differ from the placebo [37]. The underlying mechanism for this observation seems to be the strong (50%) downregulation of the ABCA1 transporter mRNA and membrane protein expression at higher doses of CER-001, therefore attenuating the active, regulated, and energy-dependent ABCA1 transporter-mediated cellular sterol efflux, as shown by Tardy et al. with their in vivo model with apoE^−/−^ mice [38]. It seems that high doses of HDL and CER-001 are less effective in slowing progression of atherosclerotic plaque, following a *U*-shaped dose-response curve. Tardy et al. concluded that at a dose of 2–5 mg/kg CER-001 could achieve maximal efficacy in removing cholesterol from lipid-laden macrophages in the atherosclerotic plaque while minimizing downregulation of ABCA1 expression.

Based on these observations, the effect of a 3 mg/kg CER-001 dose was evaluated in the CARAT study (NCT02484378) [39]. A total of 301 patients with status post ACS and a baseline percent atheroma volume > 30% in proximal 10 mm in a target vessel were randomized to receive 9 weeks of treatment with either low dose of CER-001 or placebo. Although the results of the study have only been presented and not yet officially published, treatment with CER-001 did not produce a significant effect on either of the primary efficacy endpoints (i.e., percent atheroma volume and total atheroma volume, as assessed with IVUS) when compared to placebo. Therefore, we can conclude that in the setting of post ACS and with the background of contemporary treatment, treatment with CER-001 does not represent a promising strategy. Whether CER-001 could be used in other clinical settings remains still to be examined.

One such scenario is patients with familial hypoalphalipoproteinemia (FHA) as well as homozygous familial hypercholesterolemia. In two small studies with patients of these groups, repeated (over a period of 6 months) infusions of CER-001 resulted in regression of atherosclerosis, as it was assessed with 3-T magnetic resonance imaging (MRI) scans of the carotid arteries at baseline and at the end of the study [40, 41]. There is currently a phase III clinical study running (still recruiting patients), using 3-T MRI scans of the carotid artery to evaluate the effect of CER-001 infusions on atherosclerosis in FHA patients (TANGO trial, NCT02697136).

## Discussion

The therapeutic potential of targeting HDL remains to date obscure. While population studies consistently demonstrate an inverse relationship between HDL cholesterol and cardiovascular events, multiple clinical trials over the course of the last decade have failed to demonstrate any benefit from raising HDL or infusing HDL on regression of coronary atherosclerosis or on clinical events.

The large randomized controlled trials with the cholesterol ester transfer protein inhibitors produced rather disappointing results [42, 43] with the exception of anacetrapib [10]. Anacetrapib, however, is a potent agent with additional properties such as reducing levels of apolipoprotein B containing atherogenic lipoproteins; therefore, it remains unclear whether the benefit seen should be attributed to the rise of HDL cholesterol levels or the 18% relative reduction of the non-HDL cholesterol. Moreover, extended follow-up of a subset of DEFINE participants showed that it has a long terminal half-life such that low levels of anacetrapib (about 8% of apparent steady-state on-treatment trough exposures and 2% of apparent steady-state peak concentrations) were detected in blood 2.5 to 4 years after cessation of therapy [44]. Therefore, its long-term safety is questionable and it will not reach the market.

The shift to HDL mimetics as a potential therapeutic target was guided by the observation (mentioned earlier in this document) that cholesterol efflux capacity is a strong predictor for cardiovascular events, independent of the actual HDL levels [13, 14]. Interestingly, studies have shown that disruption in the cholesterol homeostasis/cholesterol efflux pathways or prolonged exposure to a hypercholesterolemic environment can influence myelopoiesis [45]. More specifically, hematopoietic stem and multipotential progenitor cells in the bone marrow and other medullary organs may be affected, leading to monocytosis. These monocytes, once they enter the atherosclerotic plaque, may possess a pre-programmed ability to induce inflammation. The physiology of the atherosclerosis is therefore so complex that the traditional cardio-centric view of physicians regarding ischemic heart disease, i.e., plaques grow in arteries until they block blood flow, causing acute coronary and other ischemic syndromes, is now considered naive. Recent research reveals complex inflammatory signaling networks that link the brain, autonomic nervous system, bone marrow, and spleen to the atherosclerotic plaque or even the infarcting myocardium [46]. Under this perspective, therapeutic options aiming to enhance cholesterol efflux pathways in myeloid progenitors, ultimately attenuating leukocyte production, seems a reasonable choice.

Therefore, it is surprising that despite the promising theoretical background and while both apoA-I Milano and CER-001 infusions produced predictable increases in cholesterol efflux capacity, they failed to translate in a favorable effect on regression of coronary atherosclerosis/coronary plaque burden, in the setting of randomized, placebo-controlled clinical trials in humans. Like the HDL cholesterol levels in the case of CETP inhibitors before, cholesterol efflux capacity induced by the currently available agents (and in the setting they have been used) appears to be an inadequate surrogate for therapeutic use. However, the role of reverse cholesterol transport in counteracting the pathogenic events leading to the formation and development of atheroma remains a valid hypothesis, as recent studies suggest. Impaired reverse cholesterol transport due to reduced hepatic scavenger receptor BI (SR-BI) function leads to increased risk of CAD despite elevation in HDL cholesterol levels [47^•^]. Whether upregulation or enhancement of SR-BI could be a novel therapeutic approach to reducing CAD risk in the general population still remains to be proven.

It should be noted that all these elegant and well conducted clinical trials (despite the negative results) have been performed in the setting of ACS. The rational is that these patients require aggressive treatment and that they would benefit the most from an intervention. However, over the last 10–15 years, the development and wide adoption of newer-generation potent statins have greatly improved our therapeutic result and the prognosis of these patients, probably leaving little space to HDL mimetic agents to be of benefit. Moreover, there is growing evidence that HDL biology has to do more with the achievement of a steady-state, slow, and continuous flux, rather than absolute levels of HDL cholesterol. Whether these agents would be more effective when administered over a longer period of time and in the setting of stable coronary artery disease, where background therapy remains largely unchanged and therefore atherogenic lipoproteins and inflammatory markers are not significantly affected, is unknown. Of note, infusion therapies are way too impractical for long term use.

Another issue to consider is whether the primary efficacy end points chosen in the clinical studies are the most appropriate ones. Pre-clinical bench models suggest that HDL induces remodeling of the atherosclerotic plaque, in terms of cellular composition, to a more stable-appearing phenotype prior to atheroma size reduction [48]. However, there is currently no validated imaging mobility, translating into clinical outcomes, that can assess such a process with sufficient precision. On the contrary, in the case of atherosclerosis imaging with IVUS, studies have associated plaque burden and its progression to adverse cardiovascular outcomes [49], therefore supporting its use as a surrogate in the evaluation of novel anti-atherosclerotic therapies. It seems unlikely that choosing a different approach in evaluating HDL mimetics would have provided a compelling reason to advance in clinical development of these agents.

## Conclusion

Recent studies using HDL mimetics in patients with acute coronary syndrome have failed to demonstrate a favorable effect on coronary atherosclerosis, therefore suggesting that this is not a promising strategy in this particular clinical setting. On the other hand, there is sufficient evidence that reverse cholesterol transport is a valid pathway to reduce the risk for CAD. Whether HDL-focused therapy can impact plaque or clinical events in the setting of contemporary therapy remains to be determined, although with each disappointing study result, the challenge increases.

## Abbreviations

ABCA1: ATP binding cassette transporter A1
ABCG1: ATP binding cassette transporter G1
ACS: Acute coronary syndrome
apoA-I: Apolipoprotein A-I
apoB: Apolipoprotein B
apoE: Apolipoprotein E
CAD: Coronary artery disease
CETP: Cholesteryl ester transfer protein
FHA: Familial hypoalphalipoproteinemia
HDL: High-density lipoprotein
IVUS: Intravascular ultrasonography
LCAT: Lecithin-cholesterol acyltransferase
LDL: Low-density lipoprotein
MRI: Magnetic resonance imaging
mRNA: Messenger RNA
QCA: Quantitative coronary angiography
sdLDL: Small dense LDL
SR-BI: Scavenger receptor BI
